# Smart metasurface with self-adaptively reprogrammable functions

**DOI:** 10.1038/s41377-019-0205-3

**Published:** 2019-10-31

**Authors:** Qian Ma, Guo Dong Bai, Hong Bo Jing, Cheng Yang, Lianlin Li, Tie Jun Cui

**Affiliations:** 1 State Key Laboratory of Millimeter Wave, Southeast University, 210096 Nanjing, China; 2 Jiangsu Cyber-Space Science & Technology Co., Ltd., 12 Mozhou East Road, 211111 Nanjing, China; 3 State Key Laboratory of Advanced Optical Communication Systems and Networks, Department of Electronics, Peking University, 100871 Beijing, China

**Keywords:** Optical physics, Physics

## Abstract

Intelligence at either the material or metamaterial level is a goal that researchers have been pursuing. From passive to active, metasurfaces have been developed to be programmable to dynamically and arbitrarily manipulate electromagnetic (EM) wavefields. However, the programmable metasurfaces require manual control to switch among different functionalities. Here, we put forth a smart metasurface that has self-adaptively reprogrammable functionalities without human participation. The smart metasurface is capable of sensing ambient environments by integrating an additional sensor(s) and can adaptively adjust its EM operational functionality through an unmanned sensing feedback system. As an illustrative example, we experimentally develop a motion-sensitive smart metasurface integrated with a three-axis gyroscope, which can adjust self-adaptively the EM radiation beams via different rotations of the metasurface. We develop an online feedback algorithm as the control software to make the smart metasurface achieve single-beam and multibeam steering and other dynamic reactions adaptively. The proposed metasurface is extendable to other physical sensors to detect the humidity, temperature, illuminating light, and so on. Our strategy will open up a new avenue for future unmanned devices that are consistent with the ambient environment.

## Metamaterials: Sensing surfaces that program themselves

A metamaterial surface developed by researchers in China automatically adjusts its electromagnetic properties in response to changes in its environment. Metasurfaces are designed with tiny structures arranged in unique ways to manipulate electromagnetic radiation, and efforts are being made to integrate them with digital coding to produce adaptive, programmable systems. Tie Jun Cui at Southeast University in Nanjing and co-workers have designed smart metasurfaces with integrated sensors that detect features such as movement, light or temperature, linked to a microcontroller which responds by changing the configuration of a metasurface. A potential application of the system would be to help moving airplanes maintain contact with satellites; a gyroscope sensor detects changes in orientation, and the metasurface adapts its electromagnetic properties to ensure that the communication beam will always point in the satellite’s direction.

## Introduction

During the past few decades, metamaterials have attracted great interest owing to their remarkable electromagnetic (EM) properties^1–6^, which are mainly introduced by their subwavelength structures and functional arrangements. As forms of planar metamaterials, metasurfaces not only overcome the challenges encountered in bulk metamaterials (e.g., high loss and difficult fabrication^7^) but also impose strong manipulations of EM waves via, e.g., wavefront shaping^8,9^, radiation control^10–12^, and polarization conversion^13,14^. Owing to this versatility, various applications of metasurfaces have been proposed, including imaging^15–17^, invisibility and illusion^18–20^, and anomalous reflection and refraction^21,22^. Nevertheless, the abovementioned works mainly focused on continuous modulations on metasurfaces.

To explore new perspective of metasurfaces, the concepts of digital-coding metasurfaces and programmable metasurfaces were proposed^23^, establishing a link between metasurface physics and digital information science. From this viewpoint, a metasurface is characterized by digital elements ‘0’ and ‘1’ with opposite phases for 1-bit coding or digital elements ‘00’, ‘01’, ‘10’, and ‘11’ with a 90° phase difference for 2-bit coding^23^, instead of using the effective medium parameters (e.g., permittivity, permeability, and refractive index). Meanwhile, the design methods for the digital-coding metasurface can also be extended from physical principles to more general ones, including physics, information science, and digital signal processing technology. Using the new methodology, various digital-coding metasurfaces with diverse functions have been presented^24–31^. For example, the information entropy of a coding metasurface was introduced to estimate the information quantity carried by the metasurface^24^; some digital signal processing methods, such as convention theorem^25^ and addition theorem^26^ have been applied to digital-coding metasurfaces. Another hallmark achievement is programmable hologram and imaging based on the programmable metasurface, which has enabled the dynamic control of holographic images and moving-target imaging in real time^27,28^. In addition, multiple schemes of coding metasurfaces have demonstrated that information can be encoded in orthogonal orbital-angular-momentum modes and polarizations^29,30^. Some metasurfaces with similar concepts have also been proposed to realize adaptive control by human beings^32,33^. Based on shape memory materials^34,35^, a tunable metasurface was presented with a specific temperature stimulus^36^. However, the above-mentioned tunable, adaptive, and programmable metasurfaces must be controlled by human beings.

Here, we propose a smart digital-coding metasurface with self-adaptive capabilities, in which the reprogrammable functions are controlled by the metasurface itself, instead of by a human being, by using specific feedback modulations for spatial positions and other alterations. Integrated with a gyroscope sensor and an intelligent control system with a fast feedback algorithm, the presented metasurface can truly achieve sensing and automatic responses and is capable of realizing self-adaptively reprogrammable functions without the aid of human beings. The metasurface platform is also open and easily applied to other sensors, promising more elegant sensing-feedback mechanisms. We envision that this work will pave the way towards intelligent and cognitive metasurfaces.

## Results

### Principle and design

First, we clarify several concepts of active metasurfaces. Tunable metasurfaces usually realize similar functions by tuning the active devices^36–38^. Reconfigurable metasurfaces can exhibit significantly different functions by switching the active devices, but the number of functions is very limited^39–42^. Programmable metasurfaces are accompanied by digital coding characterization, in which the EM responses are manipulated by the digital-coding sequences^23,27,28^. With the aid of a field programmable gate array (FPGA), which stores a large number of coding sequences and the corresponding EM responses, a single programmable metasurface can reach many significantly distinct functions in real time by giving instructions to the FPGA^23,27,28^. In the tunable, reconfigurable, and programmable metasurfaces, however, human beings must participate in the control actions to tune and switch the active devices or send instructions to the FPGA. The reason for this participation is that all the above-mentioned metasurfaces are open-loop systems that do not contain sensing-and-feedback components to establish a smart system for automatic decision-making.

**Fig. 1:**
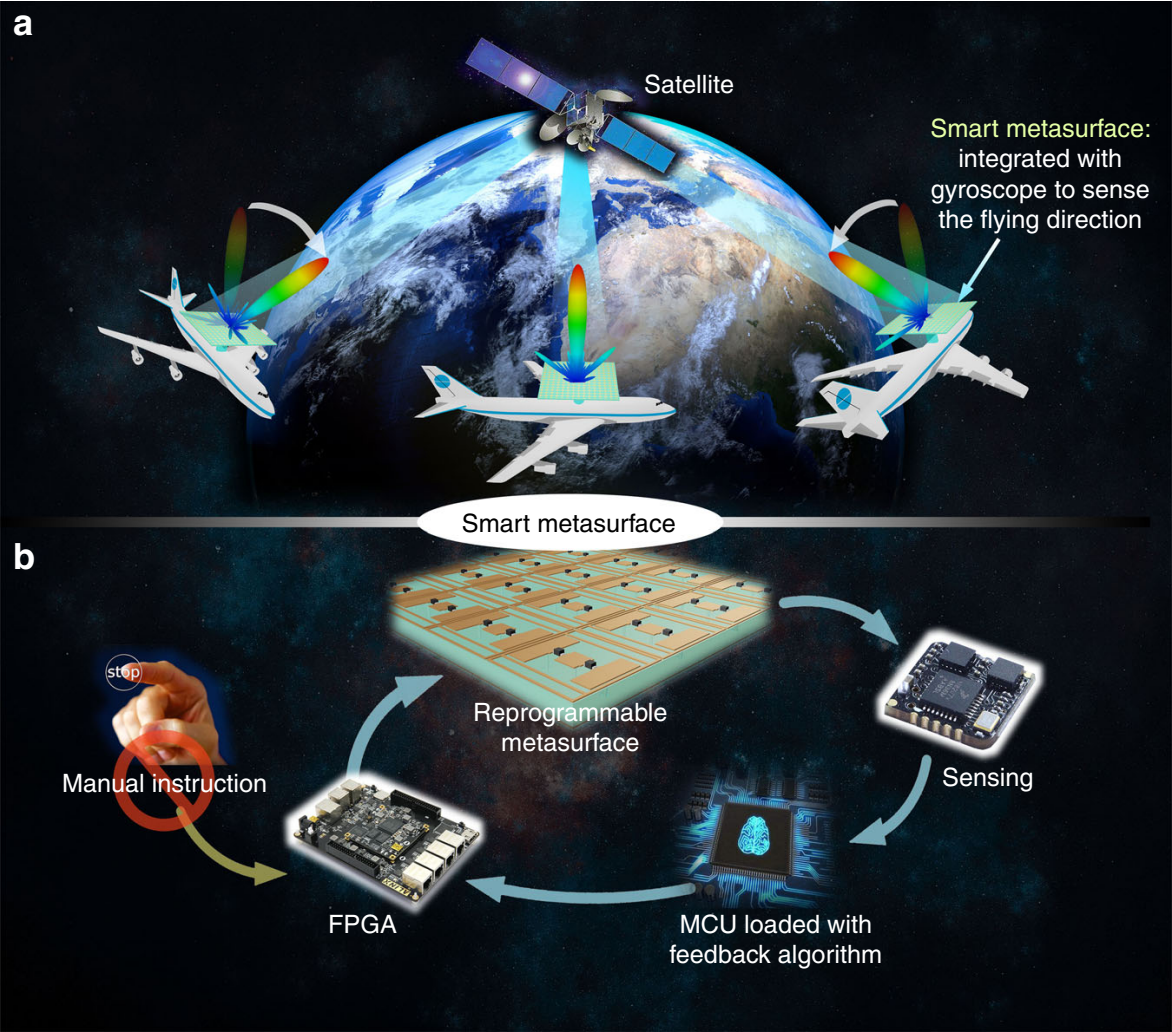
The schematic of a smart metasurface. **a** Illustration of the proposed smart metasurface with the self-adaptively reprogrammable functions without manual instruction. **b** The closed-loop system of the smart metasurface, which includes a digital-coding metasurface, an FPGA, a sensor, and an microcontroller unit (MCU) loaded with the fast feedback algorithm

Here, we propose a smart metasurface with self-adaptively reprogrammable functions, as shown in Fig. 1a. The smart metasurface is based on the programmable metasurface but consists of sensing-and-feedback components (see Fig. 1b), which help constitute a smart system for the metasurface without the need of manual instructions. The sensor on the metasurface detects some specific features of the metasurface and its environment (e.g., spatial attitude, movement status, and temperature) and sends the feature variations to a microcontroller unit (MCU). Loaded with a feedback algorithm, the MCU can determine the reaction to these variations by itself and instruct the FPGA to change the metasurface configuration (i.e., coding patterns) in real time. Thus, the metasurface realizes smart beam modulations automatically under these varied features based on the on-site calculations or precalculated database stored in the control unit. Hence, the smart metasurface can achieve self-adaptively reprogrammable functions automatically using the sensing-feedback system and calculation software on the metasurface. Owing to the excellent compatibility of the MCU, a variety of sensors can be easily integrated, which allows the smart metasurface to sense with more degrees of freedom, and more interesting functions can be realized based on different sensors.

### Smart beam manipulation

We consider a specific situation in satellite communications with a flying airplane to show the working mechanism of the smart metasurface, as shown in Fig. 1a. In the traditional technology, the airplane is equipped with a series of complex devices, such as beam steering units, a phase array, and a signal-processing modulator to emit signals towards the satellite direction, which require high cost and high complexity. However, we can replace these devices with a simple smart metasurface consisting of a gyroscope sensor and an MCU, as illustrated in Fig. 1b. Since the radiation pattern is determined by the coding sequence on the metasurface, the smart metasurface is able to adjust the coding sequence in real time to achieve the specific radiation patterns. When the airplane changes its spatial position in the sky, the equipped gyroscope sensor instantly detects the rotation angle of the metasurface and sends a message to the MCU, which can calculate the required coding pattern in real time using a fast inverse design algorithm (see the section “Materials and methods”) for this rotation angle to ensure that the radiation beam always points in the direction of the satellite. In this scenario, the smart metasurface can self-adaptively adjust the coding pattern to steer the main beam towards the satellite no matter how the metasurface is rotated. This rotating angle is equivalent to the rotated scattering-beam direction (see Supplementary Note S1). Consequently, for beam-direction steering (like beam-staring and scanning), the key task of the algorithm is to calculate the coding pattern rapidly for an arbitrary scattering direction (*θ*, *φ*). According to the generalized Snell’s law, the arbitrary scattering angle *θ* can be obtained either by a coding sequence with a specific period *n* or by combining two or more sequences with different periods (see Supplementary Note S2). In the presented inverse design algorithm, we first calculate the sequence period *n* according to the desired scattering angle *θ*; then align the detailed coding sequence based on the calculated period *n*; and finally obtain the whole coding pattern for the metasurface.

**Fig. 2:**
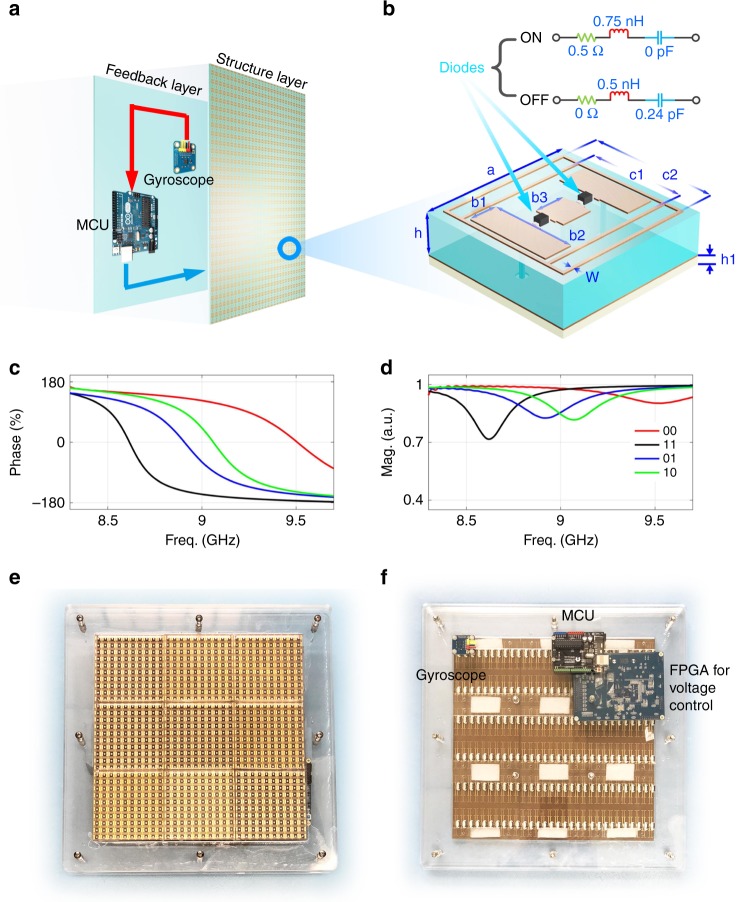
The structure and performance of the designed 2-bit reprogrammable metasurface. **a** The configuration of the proposed smart metasurface. **b** The detailed unit structure of the 2-bit digital coding metasurface. **c** and **d** The phase and amplitude responses of the 2-bit digital coding metasurface, with different colors used to indicate four digital states. **e** and **f** The front and back views of the fabricated metasurface

In our design, the sensor and microcontroller written with the fast inverse design algorithm are integrated into the back of a programmable metasurface, as illustrated in Fig. 2a. A 2-bit digital element containing two PIN diodes (SMP1320 from SKYWORKS) is proposed to construct the programmable metasurface, as shown in Fig. 2b, in which the element parameters are designed as follows: *a* = 9 mm, *b*_1_ = 1.8 mm, *b*_2_ = 5.5 mm, *b*_3_ = 2.4 mm, *c*_1_ = 6.1 mm, *c*_2_ = 8.8 mm, and *w* = 0.2 mm. The dielectric substrates of the element are made of commercial printed circuit boards, in which the upper substrate is F4B, with a height *h* = 1.6 mm, dielectric constant $\varepsilon _{\mathrm {r}} = 2.65$, and tangent loss $\tan \delta = 0.001$, and the lower substrate is FR4, with a height *h*_1_ = 0.5 mm, dielectric constant $\varepsilon _{{\mathrm {r1}}} = 4.4$, and loss tangent $\tan \delta = 0.02$. A metal sheet is inserted between the two dielectric substrates. The equivalent circuits of the PIN diode in the “on” and “off” states are given in Fig. 2b, which are used in the field–circuit joint simulations.

The two diodes in each element generate four digital states “00”, “01”, “10”, and “11”, whose simulated results of the reflection phases and amplitudes are presented in Fig. 2c, d. At the operating frequency (9 GHz), we observe that the reflection amplitudes in all states are above 0.8, which guarantees good reflection efficiency. We also observe that the phase responses of the four digital states are 123.8°, 42.8°, −58.7°, and −155.5°, respectively. Clearly, the phase difference between adjacent states is almost 90°, making the elements constitute a 2-bit digital metasurface. The fabricated smart metasurface sample is shown in Fig. 2e, f, in which the gyroscope sensor, MCU and FPGA are fixed onto the back of the metasurface.

**Fig. 3:**
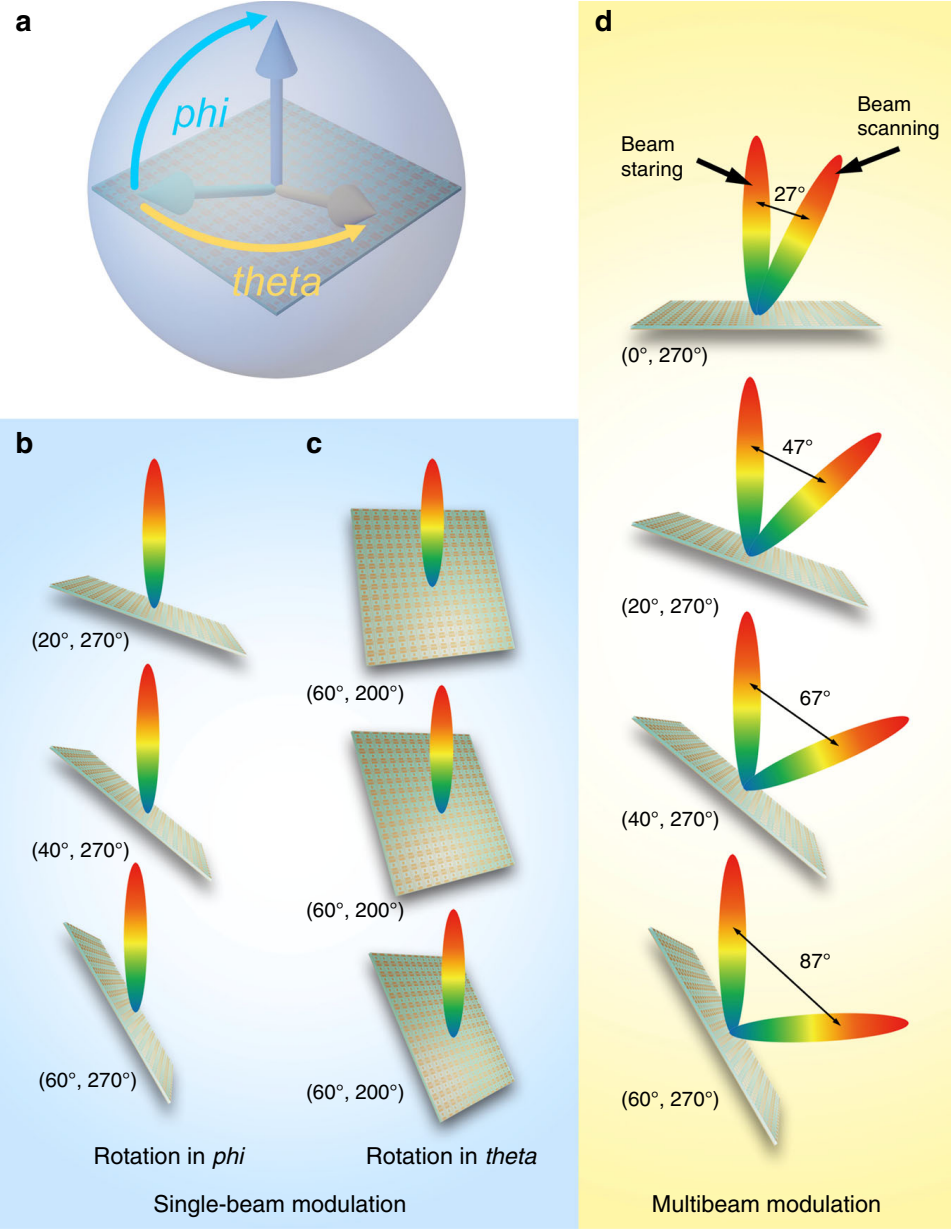
The illustration of two schemes for the spatial self-adaptive principle. **a** The illustration for Scheme A: beam steering. **b** The situations where the metasurface rotates by different elevation angles (*φ*), namely, 20°, 40°, and 60°, in which the azimuth angle is fixed at 270°. **c** The situations where the metasurface rotates by different azimuth angles (*θ*), namely, 200°, 220°, and 240°, in which the elevation angle is fixed at 60°. **d** The situations for multibeam modulation. When the metasurface rotates, one beam stares at 0°, and the other beam rotates with respect to the metasurface from 0° to 60°

To elucidate the performance of the self-adaptive controls to radiation beams by the smart metasurface, two representative schemes are presented, the coding patterns of which are calculated by using the aforementioned algorithm. To clearly exhibit the calculation process, we provide a detailed example in the “Materials and methods” section. Scheme A, shown in Fig. 3a–c, exhibits automatic single-beam steering to the north-pole direction (i.e., pointing to the satellite) no matter how the metasurface rotates along the elevation angle (*φ*) or along the azimuth angle (*θ*), as shown in Fig. 3b, c in detail. Note that the relative position of the feeding source and metasurface remains unchanged when rotating. Hence, we can easily calculate the required digital-coding sequences based on a horizontally placed metasurface with relevant deflection beams. Six rotation angles are selected: (270°, 20°), (270°, 40°), (270°, 60°), (200°, 60°), (220°, 60°), and (270°, 60°); these demonstrate that the metasurface adjusts its radiation beam self-adaptively to point to the satellite when it is at different elevations and azimuth angles (see Fig. 3b, c). In Scheme B, the smart metasurface can also realize multibeam modulations based on the fast algorithm in the MCU. We present dynamic dual-beam steering when the metasurface rotates, as depicted in Fig. 3d. We show that two radiation beams can be modulated independently using the fast algorithm. When the metasurface rotates from 0° to 60°, one beam is always directed towards the north pole (beam staring), while the other beam rotates with the metasurface to realize beam scanning, where the included angle between two beams changes from 27° to 87°.

### Simulated and experimental results

**Fig. 4:**
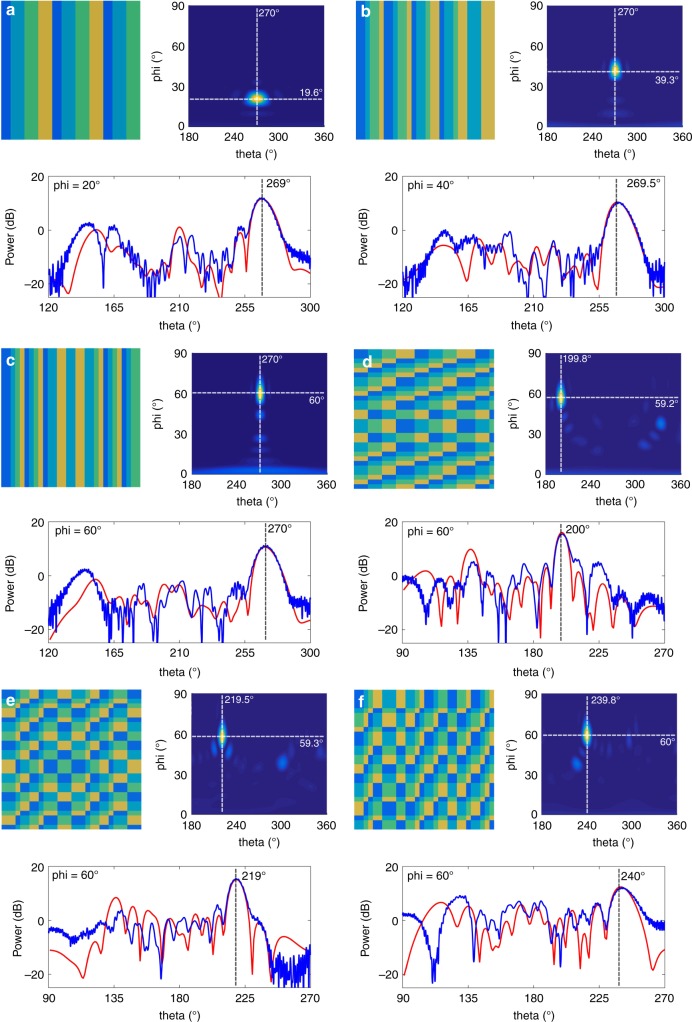
The designed digital-coding patterns and the simulated and experimental results for Scheme A, in which the simulated far-field results of the upper half space for the six situations are listed next to the coding patterns; the comparisons between the simulated and experimental far-field results for the six situations are listed below the coding patterns. Here, the simulated and experimental far-field results are marked with red and blue, respectively. **a**–**c** The three states of rotation in *φ*, with the deflecting beams at elevation angles of 20°, 40°, and 60°, in which the azimuth angle is fixed at 270°. **d**–**f** The three states of rotation in *θ*, with the deflecting beams at azimuth angles of 200°, 220°, and 240°, in which the elevation angle is fixed at 60°

For experimental demonstrations, we design and fabricate a smart digital-coding metasurface composed of 30 × 30 elements. The designed coding patterns for Scheme A in the six situations are illustrated in Fig. 4a–f. For each situation, the simulated far-field result in the upper half space is given next to the coding pattern, and the measured far-field radiation pattern is illustrated below the coding pattern. Here, the four colors in the coding patterns indicate the four designed phase responses (0°, 90°, 180°, and 270°) listed in Fig. 2c, as shown in the color bar. We observe that the simulation and experimental results are in good agreement. Fig. 4a–c demonstrate the cases where rotation is carried out along the *φ* direction, namely, *φ* = 20°, 40°, and 60°, in which the measured main beams are directed to 269°, 269.5°, and 270°, respectively, being in excellent agreement with the design value of 270°. Here, we use the aperture efficiency^43^ to evaluate the overall efficiency of the metasurface, which includes both the radiation efficiency (related to the loss from the diodes) and the illumination efficiency (related to the spillover power). The aperture efficiency of the single-beam field is ~47.2% when the deflection angle is 20°, which can be further improved by applying lower-loss PIN diodes and optimizing the phase pattern and feeding source^44^. Similarly, to verify the rotations along the *θ* direction, the test plane is always set as *φ* = 60°. Fig. 4d–f illustrate three situations: *θ* = 200°, 220°, and 240°, respectively. We note that the measured deflection angles are 200°, 219°, and 240°, which are in very good agreement with the designs. The deviation between simulations and measurements is mainly due to the following reasons: (1) an imperfect printed circuit board manufacturing process; (2) the error of manual operations in the measurement setup; and (3) nonideal plane-wave illumination.

**Fig. 5:**
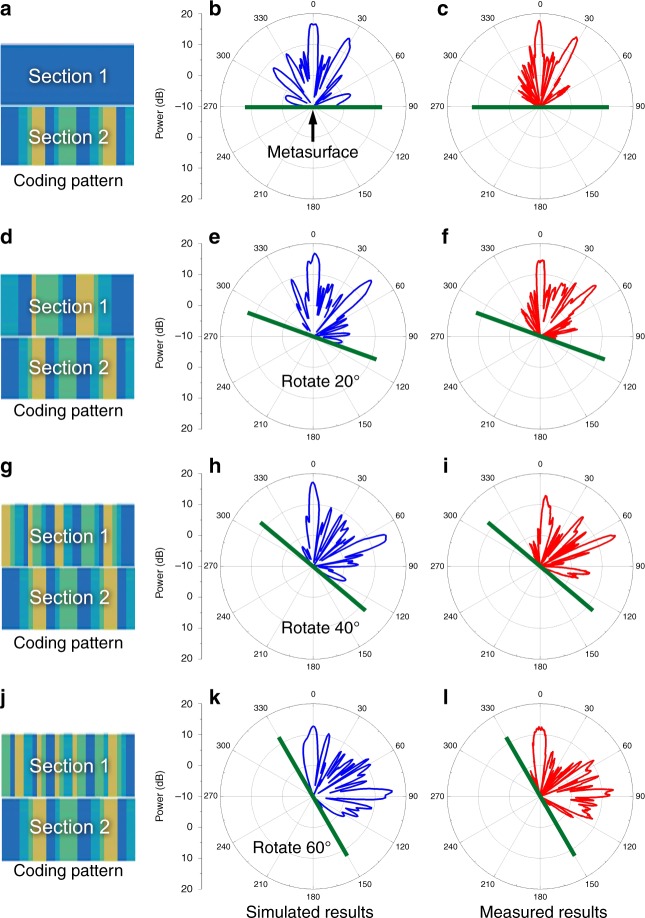
The multiple-beam smart manipulation. **a**, **d**, **g**, and **j** The calculated coding patterns for different rotation states. **b**, **e**, **h**, and **k** The simulated far-field results when the metasurface rotates from 0° to 60°. **c**, **f**, **i**, and **l** The measured far-field results when the metasurface rotates from 0° to 60°

To demonstrate Scheme B for multibeam modulations, we divide the smart metasurface into two sections. When different coding sequences are applied in these two sections, the metasurface can independently control the scattering beams corresponding to the two sections. Four groups of the calculated coding sequences are listed in Fig. 5a, d, g and j. When the metasurface rotates from 0° to 60°, the left beam is always directed to 0°, but the right beam steers from 27° to 87°. The simulated results are illustrated in Fig. 5b, e, h and k, which are marked in blue, while the measured results are presented in Fig. 5c, f, i and l. Much consistency is observed between the simulations and measurements. In the scattered fields, one beam direction varies at approximately 27°, 47°, 67°, and 87°, while the other beam always points to 0°. The slight errors between the simulations and measurements mainly come from the nonideal fabrication and manual operations.

### Smart metasurface platform

**Fig. 6:**
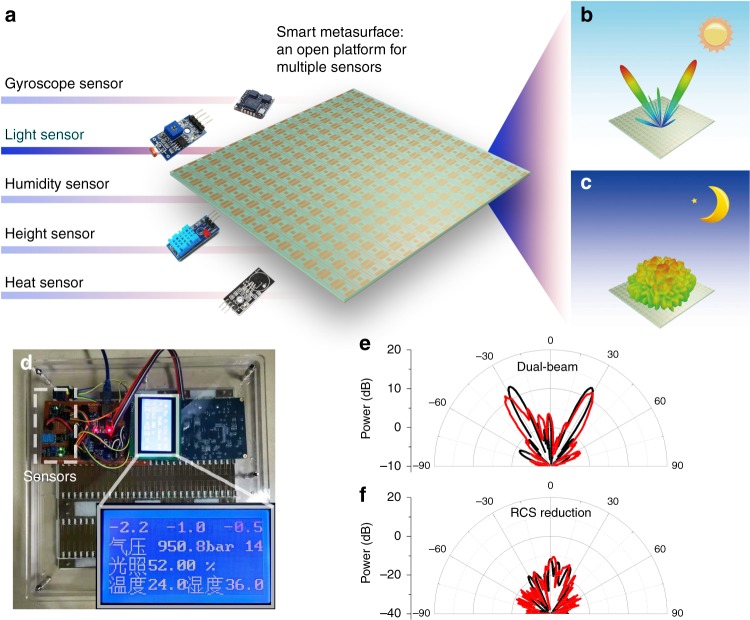
An illustration of the smart metasurface platform. **a** The smart metasurface integrated with multiple sensors. **b** and **c** The distinct reactions according to the light sensor: dual-beam radiation for the light state and RCS reduction for the dark state. **d** A photograph of the assembled smart metasurface. **e** and **f** The simulated and measured results for dual-beam radiation and RCS reduction

After integration with the MCU, the smart metasurface has a large expanding capacity for various sensing functions. Not only the gyroscope but also other sensors can be embedded. Our metasurface is a smart platform onto which multiple sensors can be connected, as shown in Fig. 6a. With these different sensors, the smart metasurface is able to detect and react to more kinds of variation. Here, we take a light sensor (GL5516) as an example. The light sensor can detect the intensity of visible light (~380–780 nm) and produce an intensity percentage. For different light intensities, the metasurface can be programmed to generate dual-beam radiation or diffuse scattering for light or dark illumination, as depicted in Fig. 6b, c. Using this sensor, we successfully combine the visible-optical stimulus with microwave radiation.

To experimentally validate our design, five kinds of sensors, namely, a gyroscope, light sensor, humidity sensor, height sensor, and heat sensor, are integrated into our smart platform, as shown in Fig. 6a. The assembled metasurface platform is exhibited in Fig. 6d. A display screen is also embedded on the back of the metasurface to show the real-time states of these sensors. The detailed information displayed on the screen is provided in Supplementary Information Note S9. For the light sensing-reaction demonstration, we simulate and measure two radiation patterns: dual-beam radiation and radar cross section (RCS) reduction, as illustrated in Fig. 6e, f, in which the simulated and measured results are marked by the red and blue lines, respectively. Good agreement is observed between the simulations and experiments.

## Discussion

**Fig. 7:**
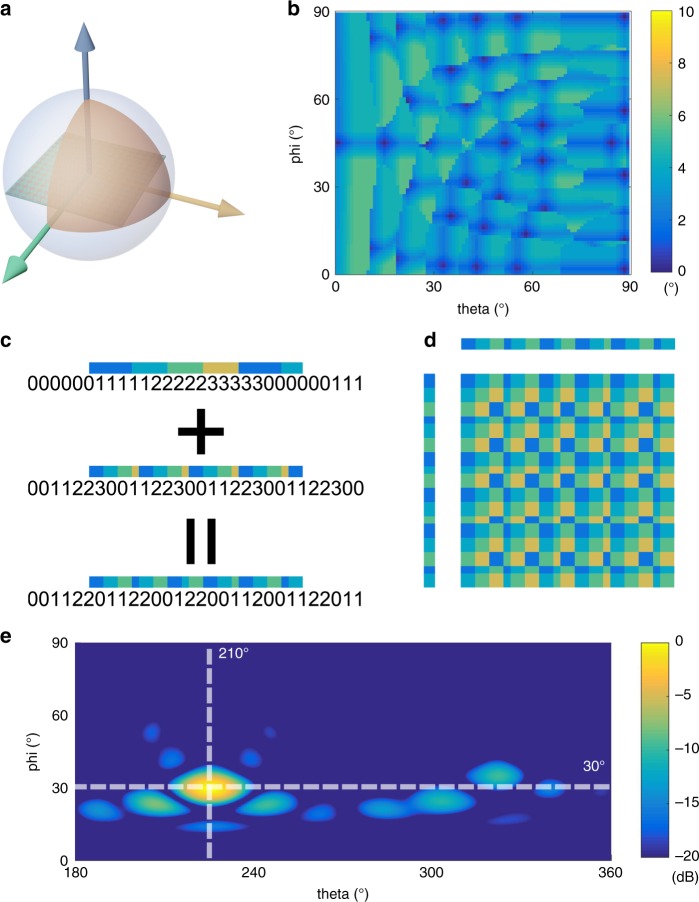
The coding pattern calculation process and its design error analysis. **a** The illustration for beam deflections in the first quadrant. **b** The error angle distribution for beam deflections in the first quadrant (*θ* and *φ* vary from 1° to 90°). **c** The calculation process for the digital-coding sequence. **d** The calculated digital-coding pattern. **e** The simulated far-field result in the upper half space

From the above analyses, the smart metasurface is determined by three main factors: the digital and programmable metasurface with the FPGA, the sensor, and the fast algorithm for the inverse design of coding patterns, in which the third factor is very important for the whole system. To demonstrate the design accuracy of the algorithm, we exhibit the error distribution for beam deflections in the upper half space, in which the error is calculated as the absolute value of the difference between the target and calculated angles (see Supplementary Information Note S4 for details). Owing to the symmetry of the coding pattern, the beam deflection in one quadrant can be transferred to the other three quadrants. Hence, we need to analyze only the 2D coding patterns for the beam deflections in the first quadrant shown in Fig. 7a. The error distribution of the target direction in the first quadrant is presented in Fig. 7b, where both *θ* and *φ* vary from 0° to 90°. From this figure, it is obvious that the error angles are almost below 5° and even below 1° in many areas, implying very good performance. For more details regarding the calculation process and results, please refer to Supplementary Information Notes S1–S4.

In the main results, we have studied automatic single-beam and multiple-beam modulations using the smart metasurface. If the application scenario becomes more complicated, it is difficult to calculate the desired coding patterns for different features in real time. In this case, we can precalculate the coding patterns and store them in the MCU to realize the required reprogrammable functions adaptively. For example, when we design the smart metasurface to achieve dynamic reactions, such as generating orbital angular momentum (OAM) beams and reducing the RCSs under different rotation angles of the metasurface, as shown in Fig. S12, we calculate the coding patterns corresponding to the rotation angles and set up a database stored in the MCU, which is used to reach the required functions automatically in real time. In fact, diverse functionalities can be designed by programming multiple algorithms in the MCU. The numerical simulations and experimental verifications are presented in Supplementary Information Note S10 and Fig. S12.

In summary, we proposed a new smart metasurface concept, namely, a self-adaptive digital-coding metasurface, which contains a complete sensing and feedback mechanism. To illustrate our idea, we presented a specific smart metasurface to realize self-adjustment of the radiation beams by the metasurface itself in real time for spatial variation without human control. Integrated with a gyroscope sensor and fast feedback algorithm, we demonstrated automatic singlebeam and multibeam modulations when the metasurface rotates. More sensors were equipped on the smart metasurface, such as a light sensor, which successfully connects the optical stimulus and microwave modulations. A 2-bit digital-coding smart metasurface in the X band was designed, fabricated, and measured. The experimental results were in good agreement with the numerical simulations, validating the adaptive sensing-feedback schemes. The presented smart metasurface can be further extended to higher frequencies by using nanostructures with MEMS^45^ or liquid crystal^46^. As illustrated in the work, the smart metasurface is composed of three main parts: programmable units, an FPGA, and sensors. These parts are used to construct the sensing-feedback system. In this architecture, the sensor needs only to be fixed onto the edge of the metasurface, and thus, it has no more interference with the metasurface units. We remark that various smart metasurfaces can be realized as desired by equipping relevant sensors. The proposed concept offers a new definition of a metasurface, which also paves the way towards cognitive and intelligent metasurfaces^45–49^.

## Materials and methods

### Fast feedback algorithm

Different from the conventional metasurface design, in which the field calculation $f\left( {\theta ,\varphi } \right)$ is based on the two-dimensional (2D) coding pattern P(*x*, *y*) (see Supplementary Note S1), the coding pattern here is in the three-dimensional (3D) space P(*x*, *y*, *z*). Therefore, the field expression becomes $f\left( {\theta + \Delta \theta ,\varphi + \Delta \varphi } \right)$ for the same coding pattern in the 2D situation. For beam steering, the pattern calculation of the metasurface is equivalent to finding a pattern with beam deflection $( - \Delta \theta , - \Delta \varphi )$ in a 2D plane. According to the convolution operations of the digital-coding metasurface^24^, the scattering direction of the coding metasurface in spherical coordinates (*θ, φ*) is derived as

1 $$\left\{ {\begin{array}{*{20}{l}} {\theta = {\mathrm {sin}}^{ - 1}(\sqrt {{\mathrm {sin}}^2\theta _x \pm {\mathrm {sin}}^2\theta _y} )} \\ {\varphi = {\mathrm {tan}}^{ - 1}({\mathrm {sin}}\theta _x/{\mathrm {sin}}\theta _y)} \end{array}} \right.$$

in which *θ*_*x*_ and *θ*_*y*_ are the deflection angles of the coding sequences grading along the *x*- and *y*-directions, respectively. To approach the design value as closely as possible, the coding sequence can be further recalculated as

2 $$\theta _x = {\mathrm {sin}}^{ - 1}({\mathrm {sin}}\theta _{x1} + {\mathrm {sin}}\theta _{x2})$$

in which $\theta _{x1}$ and $\theta _{x2}$ are deflection angles of the two gradient coding sequences. From Eq. (2), multiple deflection angles can be obtained with finite gradient coding sequences. The detailed calculation process is provided in Supplementary Note S2.

To further illustrate the inverse design algorithm, we provide a calculation example for a target deflection angle (30°, 45°). According to Eq. (1), we first resolve the target angle into ${\mathrm {sin}}\,\theta _x$ and ${\mathrm {sin}}\,\theta _y$, which can be easily obtained by ${\mathrm {sin}}\theta _x = {\mathrm {sin}}\theta _y = \frac{{\sqrt 2 }}{2}$. Then, by applying Eqs. (S7)–(S10) (see Supplementary Note S2), we obtain two arrays *n*_1_ and *n*_2_ that include the possible values of the element number. We substitute *n*_1_ and *n*_2_ into Eqs. (S5) and (S6) and solve ${\mathrm {sin}}\,\theta _x$ and ${\mathrm {sin}}\,\theta _y$ as

S11 $${\mathrm {sin}}\,\theta _x = {\mathrm {sin}}\,\theta _y = \frac{{\sqrt 2 }}{4} \approx \frac{\lambda }{P}(\frac{1}{{25}} + \frac{1}{{18}})$$

Therefore, the coding sequence along the *x*- and *y*-axis can be obtained by adding two gradient series with periods of 25 and 18. These two gradient series can be expressed as “0000000111111222222333333” and “000001111122223333”, which should be prolonged to “000000011111122222233333300000” and “000001111122223333000001111122”, respectively. The coding sequence is finally calculated as: “000001122233301111233330011122”, as shown in Fig. 7c. As a result, the whole digital-coding pattern is calculated, as illustrated in Fig. 7d. The related far-field result in the upper half space is given in Fig. 7e, which shows good agreement with the design target.

If the coding metasurface is large enough, the angle range can be infinitely close to cover the whole upper hemispherical space. In addition, it is easy to show that a smaller element period can yield a better angle resolution. For the proposed smart metasurface, we have explored the error distribution for beam deflecting in the upper half space, as shown in Fig. 7a, b, which demonstrates good performance. It was demonstrated that most deflecting directions in the upper half space have a better resolution than 3°, and some directions have a resolution of even <1° (see Supplementary Note S4). The fast design algorithm has been written to the control unit to calculate the required deflection angle and the corresponding coding pattern under the detected rotation angle of the metasurface in real time.

### Simulation and sample fabrication

The simulation results of the unit cell and metasurface are obtained using commercial software, namely, CST Microwave Studio, with a time-domain solver. The sample is fabricated using the print-circuit board (PCB) technology. The real-time monitoring and processing of spatial variation is executed by the MCU (Arduino UNO R3), which is connected to an FPGA (for voltage control) and a gyroscope sensor (ADXL335 3-Axis). The detailed operating process of this closed-loop system is provided in Supplementary Note S5. The control components, namely, the gyroscope, MCU, and FPGA, are fixed onto the back of the smart metasurface (see Supplementary Notes S6 and S7 for details). The maximum power consumption is ~10 W, including 3.6 W for diodes (each PIN diode requires ~1 mA at 2 V), 1 W for sensors, and 5 W for the FPGA.

### Experimental measurement

The experimental setup is shown in Supplementary Fig. S10, which is used in a standard microwave chamber. The fabricated metasurface with its supporter is fixed onto a rotatable table (see Supplementary Fig. S11). A small horn antenna, with the metasurface as the feeding source, is installed 1.5 m away from the metasurface. At this distance, the radiation wave illuminated on the metasurface can be approximately regarded as a plane wave. Since the experimental system can measure only the far-field radiation results in a 2D plane, a testing section (with a certain *θ*) is selected for each situation. The status of the self-adaptive metasurface is locked in the process of measurement, which means that the coding pattern will not change, resulting in accurate testing. For more details, please refer to Supplementary Note S8.

## Supplementary information

Supplementary Information for Smart metasurface with self-adaptively reprogrammable functions
