# Supplementary material for: Smart metasurface with self-adaptively reprogrammable functions
Source: Light Sci Appl. 2019 Oct 31;8:98. doi: 10.1038/s41377-019-0205-3 (PMC6823478; doi:10.1038/s41377-019-0205-3)
Supplement: Supplementary file 1 — Supplementary Information for Smart metasurface with self-adaptively reprogrammable functions [file 41377_2019_205_MOESM1_ESM.docx]

**Supplementary Information for**

**Smart metasurface with self-adaptively reprogrammable functions**

Qian Ma^1^, Guo Dong Bai^1^, Hong Bo Jing^1^, Cheng Yang^1,2^, Lianlin Li^3^, and Tie Jun Cui^1,*^

^1^ State Key Laboratory of Millimeter Wave, Southeast University, Nanjing 210096, China

^2^ Jiangsu Cyber-Space Science & Technology Co., Ltd., 12 Mozhou East Road, Nanjing 211111, China

^3^ State Key Laboratory of Advanced Optical Communication Systems and Networks, Department of Electronics, Peking University, Beijing 100871, China

^*^ E-mail: tjcui@seu.edu.cn.

**The PDF file includes:**

- Note S1. The illustration of field calculation for rotated metasurface.
- Note S2. The illustration of the fast inverse design algorithm for coding pattern.
- Note S3. The calculation examples based on the inverse design algorithm.
- Note S4. The discussion on the accuracy of the design algorithm.
- Note S5. The detailed operating process of the smart closed-loop system.
- Note S6. The illustration for the gyroscope module and MCU.
- Note S7. The elaboration of fabricated metasurface sample.
- Note S8. The illustration of the experimental configuration.
- Note S9. The illustration of the real-time display of sensor states.
- Note S10. The coding patterns and far-field results for other functions.
- Supplementary Figures S1-S11.

**Note S1. Illustration of field calculation for a rotated metasurface**

The scattering-field calculation for the conventional metasurface generally depends on the phase distribution on a 2D plane, which can be defined as *P*(*x*, *y*) (see Supplementary Fig. S1a). For a metasurface including *N*◊*N* elements, the related scattering field is calculated as [22],

$f\left( \theta,\varphi\right)=\sum_{m=1}^{N} \sum_{n=1}^{N} \exp\left\{ -i\left\{ \varphi\left( m,n \right)+kD\sin\theta\left[ \left( m-\frac{1}{2} \right)\cos\varphi+\left( n-\frac{1}{2} \right)\sin\varphi\right] \right\} \right\}$ (S1)

where $\theta$ and $\varphi$ are the elevation and azimuth angles of an arbitrary direction. When the metasurface rotates to a certain angle, the coding pattern on the metasurface has to be extended to a 3D version *P*(*x*, *y*, *z*), as shown in Fig. S1b, and the related field is $f\left( \theta+\Delta\theta,\varphi+\Delta\varphi\right)$:

$f\left( \theta+\Delta\theta,\varphi+\Delta\varphi\right)=\sum_{m=1}^{N} \sum_{n=1}^{N} \exp\left\{ -i\left\{ \varphi\left( m,n \right)+kD\sin\left( \theta+\Delta\theta\right)\left[ \left( m-\frac{1}{2} \right)\cos\left( \varphi+\Delta\varphi\right)+\left( n-\frac{1}{2} \right)\sin\left( \varphi+\Delta\varphi\right) \right] \right\} \right\}$ (S2)

where $\Delta\theta$ and $\Delta\varphi$ are the rotations in the elevation and azimuth angles relative to the 2D version. The smart metasurface is able to detect the spatial rotation $(\Delta\theta,\Delta\varphi)$, and automatically adjust the coding pattern. For automatic beam steering, the digital coding pattern should generate additional beam deflection angles $(-\Delta\theta,-\Delta\varphi)$ to counteract this variation. The gyroscope can exactly detect this variation $(-\Delta\theta,-\Delta\varphi)$, and instructs FPGA to generate the related coding pattern. Thus the problem can be simplified to calculate the coding pattern for the specific scattering direction $(-\Delta\theta,-\Delta\varphi)$, which is perfectly solved by our inverse design algorithm for coding pattern presented in Note S2.

**Note S2. Fast inverse design algorithm of the coding pattern**

We firstly start with a gradient coding metasurface for the beam deflection in the elevation angle. The metasurface is composed of *N*◊*N* elements, and the period of each element is P. According to the generalized Snell’s law, arbitrary scattering angle *θ* can be calculated as


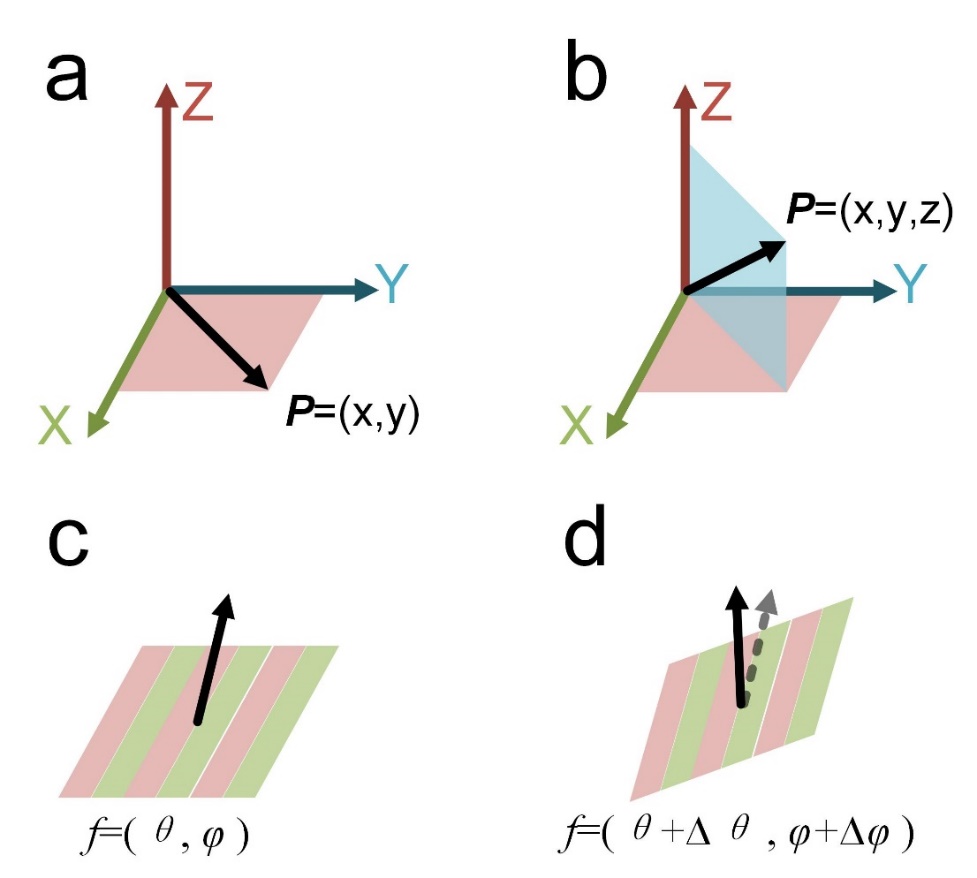


**Supplementary Figure S1.** The illustration of scattering-field calculation for rotated metasurface. (a-b) The coding patterns for 2D and 3D versions. (c-d) The 2D far-field scattering calculations.

$\theta=\sin^{-1} \frac{\lambda_{0}}{nP}$ (S3)

in which $\lambda_{0}$ is wavelength at the central frequency and *n* is the element number in a period. Eq. (S3) can also be expressed as following based on Eq. (2):

$\theta=\sin^{-1}(\frac{\lambda_{0}}{n_{1}P}\pm\frac{\lambda_{0}}{n_{2}P})$ (S4)

in which *n*_1_ and *n*_2_ are the element numbers in a period for two coding sequences. As the element number is integer, an error exists between the target angle and realistic angle. For sequence filtration, we define an error function $\mathrm{erf} \left( n \right)$ as following according to Eq. (S3) and (S4):

$\mathrm{erf} \left( n \right)=\left| \sin\theta-\frac{\lambda}{nP} \right|$ (S5)

or

$\mathrm{erf} \left( n \right)=\left| \sin\theta-\left( \frac{\lambda}{n_{2}P}\pm\frac{\lambda}{n_{1}P} \right) \right|$ (S6)

in which $n, n_{1}, n_{2}\in Z^{+}$. Hence $n_{1}$ and $n_{2}$ can be expressed as

$n_{1}=\left\lfloor\frac{2*\lambda}{sin\theta*P} \right\rfloor-i, i\in\left\{ {0, Z}^{+} \right\}$ (S7)

$n_{2}=\left\lceil\frac{2*\lambda}{sin\theta*P} \right\rceil+j, j\in Z^{+}$ (S8)

Besides, $n, n_{1}$ and $n_{2}$ should satisfy the following equations:

$\left\lceil\frac{\lambda}{P} \right\rceil\leq n_{1}$ (S9)

${n,n}_{2}\leq2N$ (S10)

The calculation target of Eq. (S5) and (S6) is to obtain the closest values of $n_{1}$ and $n_{2}$ (or $n$), in satisfying Eq. (S7)-(S10). With the best value of $n_{1}$ and $n_{2}$ (the closest value according to Eq. (S5) and (S6)), the related coding sequence is obtained, as shown in Fig. S2. For an *N*◊*N* metasurface, the length of coding sequence is *N*. When we get the best *n*, we should repeat the coding series until it is prolonged to the length *N*. In Fig. S2, *x* and *y* are the quotient and remainder by dividing *N* by *n*. Note that Eq. (S9) and (S10) limit the possible value range for${n, n_{1} and n}_{2}$. This range can be further extended, but requires much greater calculation quantity. For the applications in this work, we limit them within $\left[ \left\lceil\frac{\lambda}{P} \right\rceil, 2N \right]$ for both accuracy and calculation speed.

We remark that Eq. (S6) can be decomposed with Eq. (S4), which will further reduce the design error. For a 2-bit dogotal coding metasurface, we analyze the design error (see Note S3) with only Eq. (S5) and (S6), and find that the above calculations can achieve the accuracy of 1, which satisfies a general situation. Hence we do not need to perform more iterations to Eq. (S6) for higher accuracy.


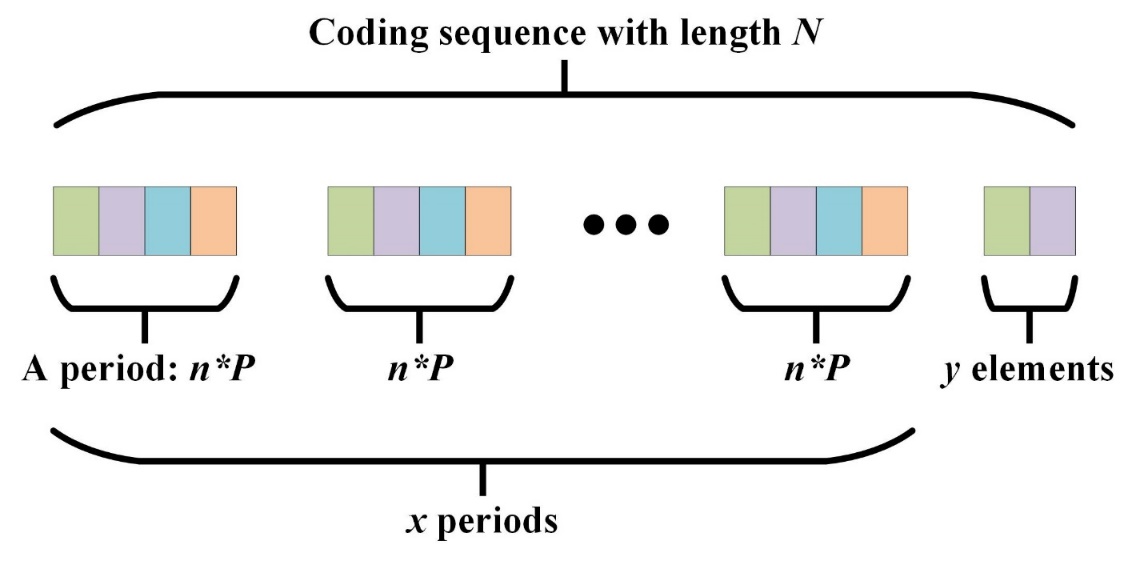


**Supplementary Figure S2.** The illustration of calculating the digital coding sequence.

**Note S3. Calculation performance** **based on the inverse design algorithm**

To further present the performance of the calculating algorithm, we provide nine deflecting angles for the coding sequences along the *x-*axis, as shown in Fig. S3, in which Fig. S3a-i demonstrate the far-field results calculated by Eq. (S1) with deflecting angles from 10º to 90º. The relevant coding sequences are given above each subgraph. These calculation results suggest great accuracy and scanning scope, illustrating good performance of this algorithm.

**Note S4. Discussion on accuracy of the design algorithm**

As mentioned above, the accuracy of the design algorithm mainly depends on the iterations of Eq. (S4) and (S6). To clearly exhibit the accuracy when using one iteration, we explore the design error for one-dimensional (1D) coding sequence and 2D coding pattern in the upper half space. For 1D coding sequences, the error distribution is listed in Fig. S4a when the target deflecting direction varies from 1° to 90°. We remark that the calculated direction shown in this figure is obtained from the designed coding sequence and Eq. (S1). It is observed that the error in most cases is below 1°, suggesting good accuracy. When the target angle is very small (close to 0°) or very large (close to 90°), the error becomes larger. According to the generalized Snell’ s law, when the deflecting angle is very small, the gradient sequence becomes very long, even far beyond the metasurface’s dimension, which contributes the main error. In the contrast, when the target angle is very large, the length of coding sequence should be very short. The predetermined size of unit


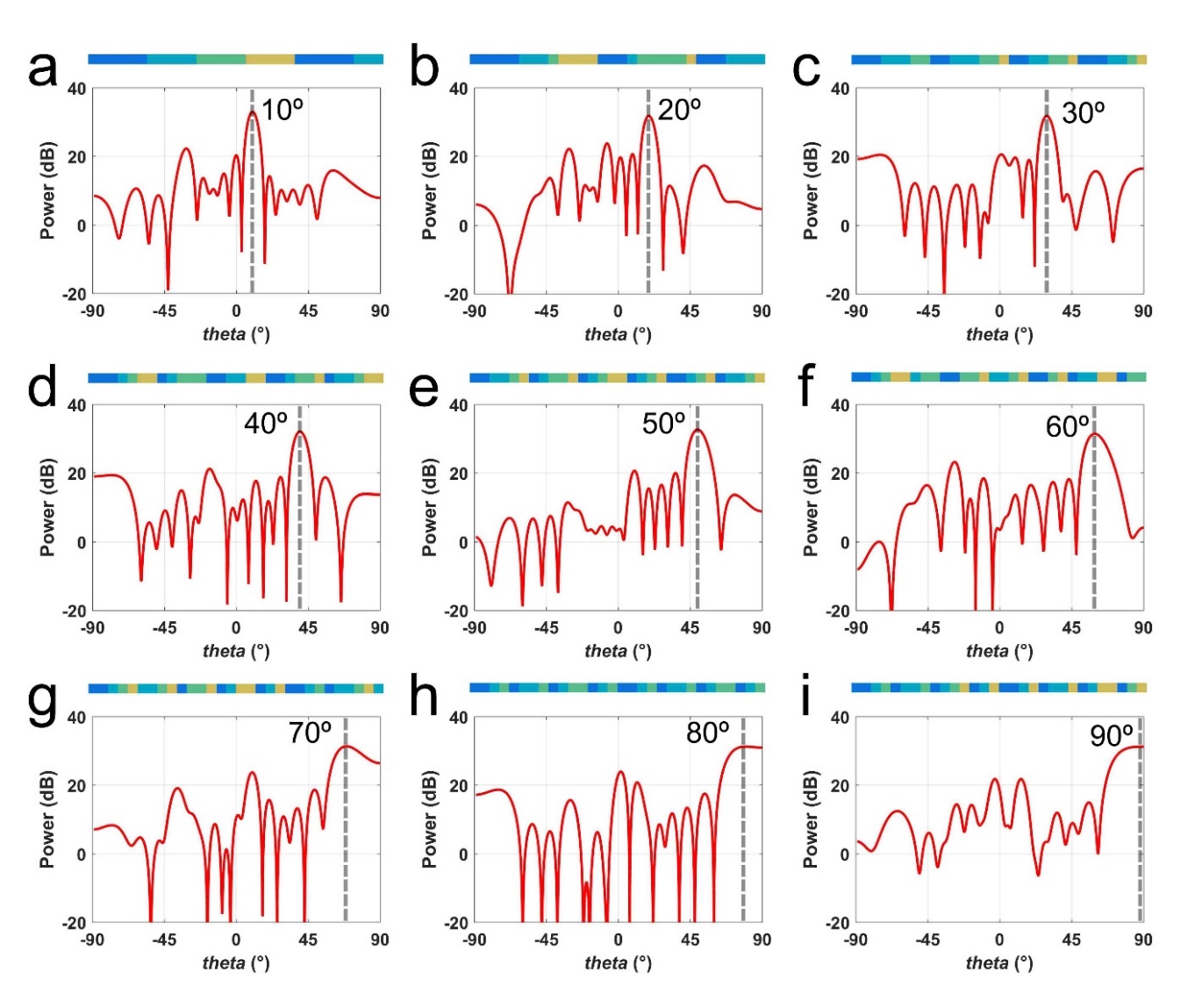


**Supplementary Figure S3.** Demonstration of inverse design algorithm for different deflecting angles. (a-i) The theoretical calculation results of far-field radiation patterns for deflecting angles from 10º to 90º using the designed coding sequences given above each subgraph. We clearly observe that the main beams are directed to the design angles.

cell limits the shortest period length and results in the large error (about 5° when the target direction angle is 85°). However, we notice that the large deflecting angles generate larger beam width, which means that the power at the target direction is also very high, as shown in Fig. S4b-S4e.


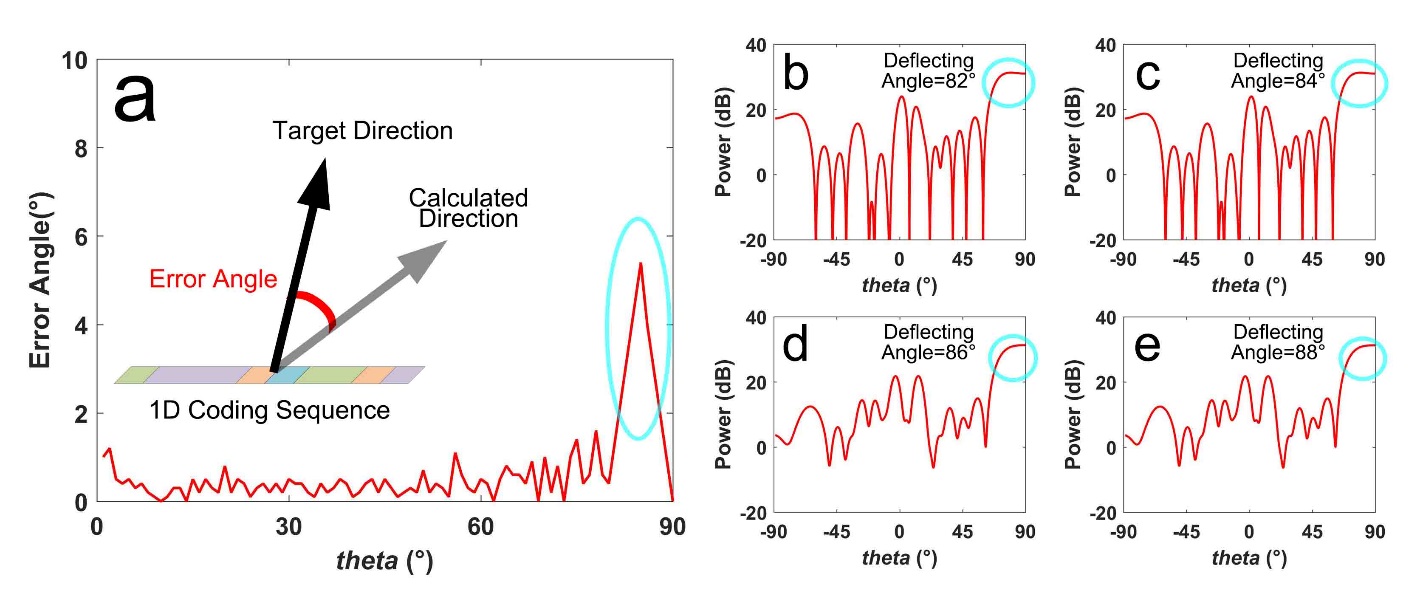


**Supplementary Figure S4.** The design error distribution for the 1D coding sequences. (a) The errors for different deflecting angles (from 1° to 90°). (b-e) The calculated far-field results when the target angle is 82°, 84°, 86°, and 88°, respectively.

**Note S5. The operating process of the smart closed-loop system**

In general self-adaptive status, the gyroscope sensor sends the rotation and attitude information to the microcontroller unit (MCU) every half second to reach real-time manipulation. The system framework is presented in Fig. S5. The gyroscope sensor sends a message including three-axis angles per half-second to MCU (Arduino UNO R3). MCU receives the message and translates it into the deflection direction $(-\Delta\theta, -\Delta\varphi)$, and then performs the reserve coding design algorithm to obtain the final coding pattern, which is sent to FPGA for diode voltage control. The final-calculated patterns are translated into bias-voltage signals (0V and 5V) in FPGA and delivered to the metasurface for diode control.


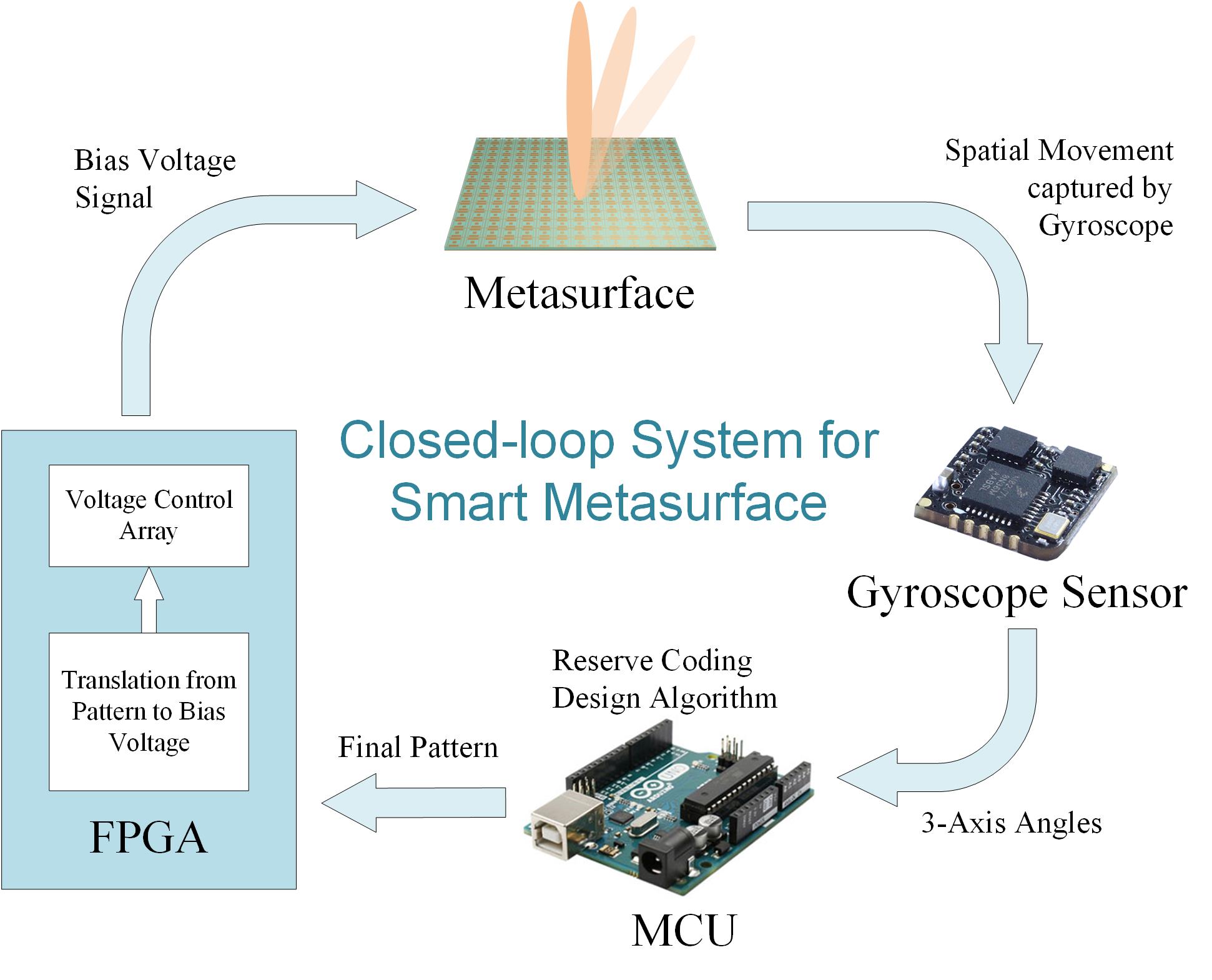


**Supplementary Figure S5.** The operating process of the proposed closed-loop system.

**Note S6. The gyroscope module and MCU**

The gyroscope and MCU applied in this work are commercial modules, ADXL335 3-Axis and Arduino UNO R3, as shown in Figs. S6a and S6b. The sensing accuracy of gyroscope is ±1° in three axes (*x*-, *y*- and *z*-axes). The detected angle is delivered into MCU within analog voltage, then is converted into digital signals. The code of the reserve coding design algorithm is loaded into MCU for pattern calculation. Arduino UNO R3 carries a microprocessor (Atmel Atmega328), which contains various I/O ports and extension potential. Both gyroscope and MCU are enabled with 5V DC supply. The working frequency of gyroscope is more than 500 Hz, promising small enough sensing period. The clock frequency of MCU is up to 16 MHz, which guarantees very fast processing speed.


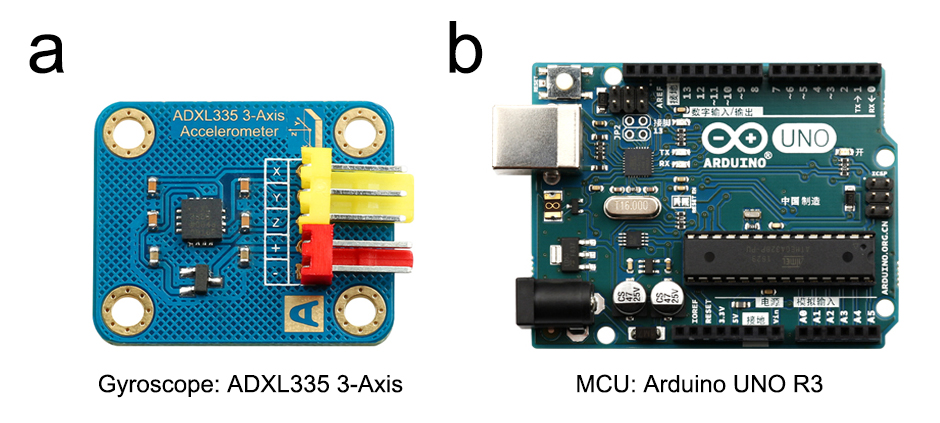


**Supplementary Figure S6.** The gyroscope and MCU applied in the smart metasurface. (a) The gyroscope module, ADXL335 3-Axis. (b) The MCU module, Arduino UNO R3.

**Note S7. The elaboration of fabricated metasurface sample**

In sample fabrication, the metasurface is integrated with 1800 PIN diodes, which can be controlled independently by an FPGA voltage control array. The gyroscope sensor (ADXL335 3-Axis) and MCU are installed on the acrylic support board on the back of metasurface. The front and back sides of the assembled smart metasurface is presented in Figs. S7a and S7b. A triaxial rotatable support is employed to hold the metasurface with screws.


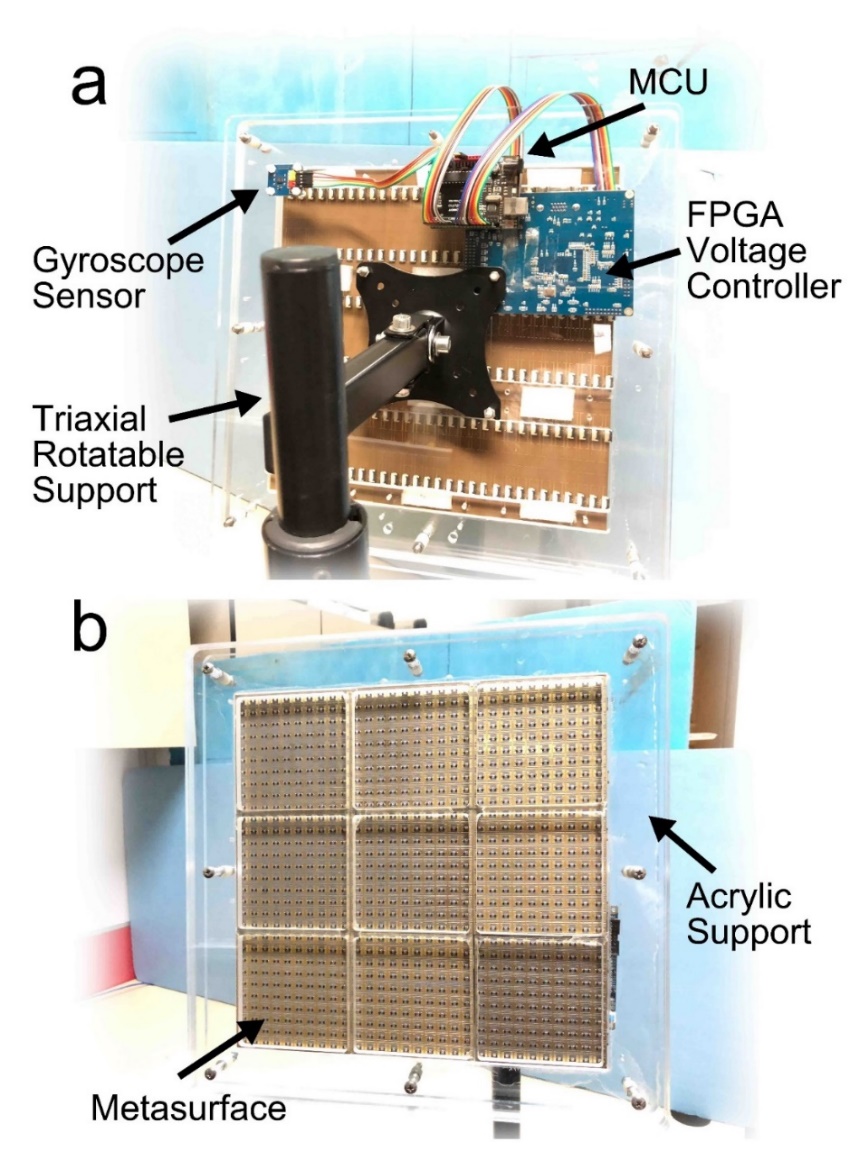


**Supplementary Figure S7.** Measurement setup and fabricated sample. (a,b) The back and front sides of the smart metasurface.

**Note S8. The experimental configuration**

In our experimental configuration, the whole system is fixed on a rotatable table for 2D far-field measurements, as illustrated in Figs. S8 and S9, in which the detailed element photograph is given in Fig. S9. The measurements are performed in a standard microwave chamber, and the operating frequency is set at 9 GHz. A small waveguide horn antenna is applied as feeding source, which moves together with the metasurface. The receiving horn is a broadband rectangular horn antenna, placed at 10 meters away from the metasurface. As the measurement system can only test the far-field power in a 2D plane, for a deflecting angle $(\theta,\varphi)$, we first rotate the metasurface in the azimuth direction, and measure this plane to verify the deflecting angle in the elevation direction.


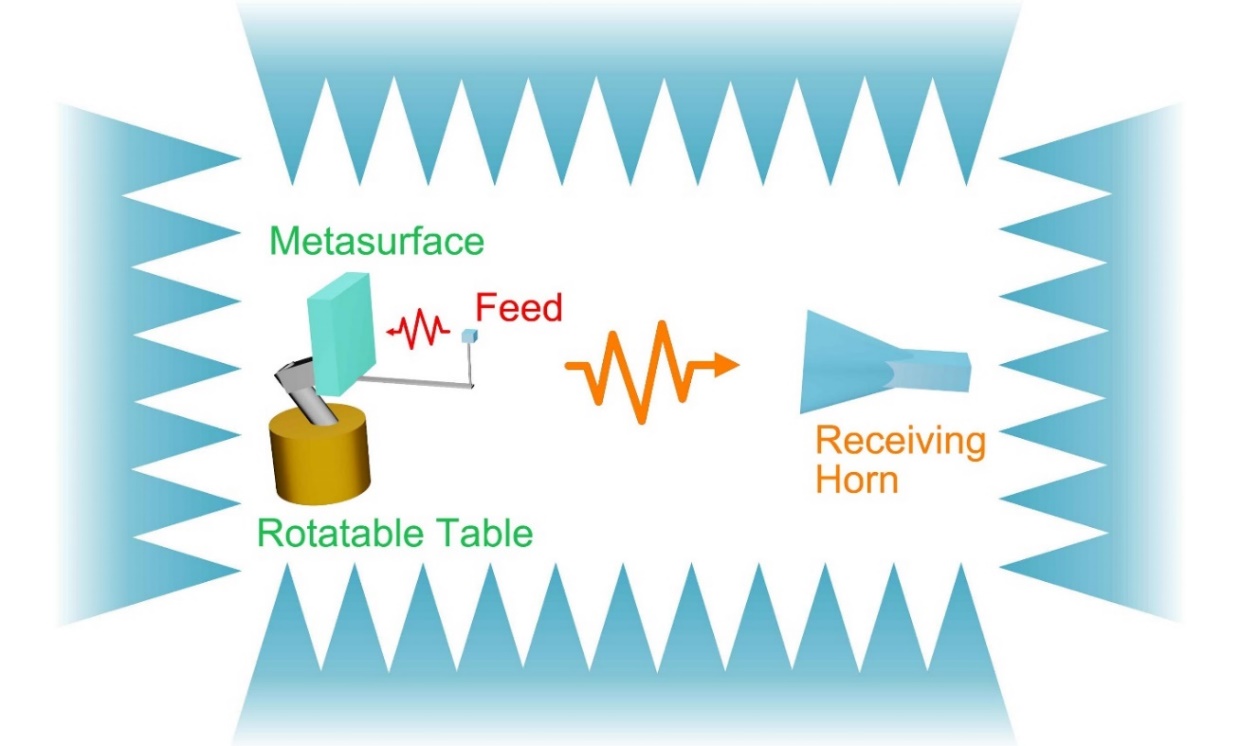


**Supplementary Figure S8.** The illustration of the experimental configuration. The measurement setup, in which the fabricated metasurface and feeding source are fixed on the rotatable table, and a receiving horn antenna measures the far-field results.


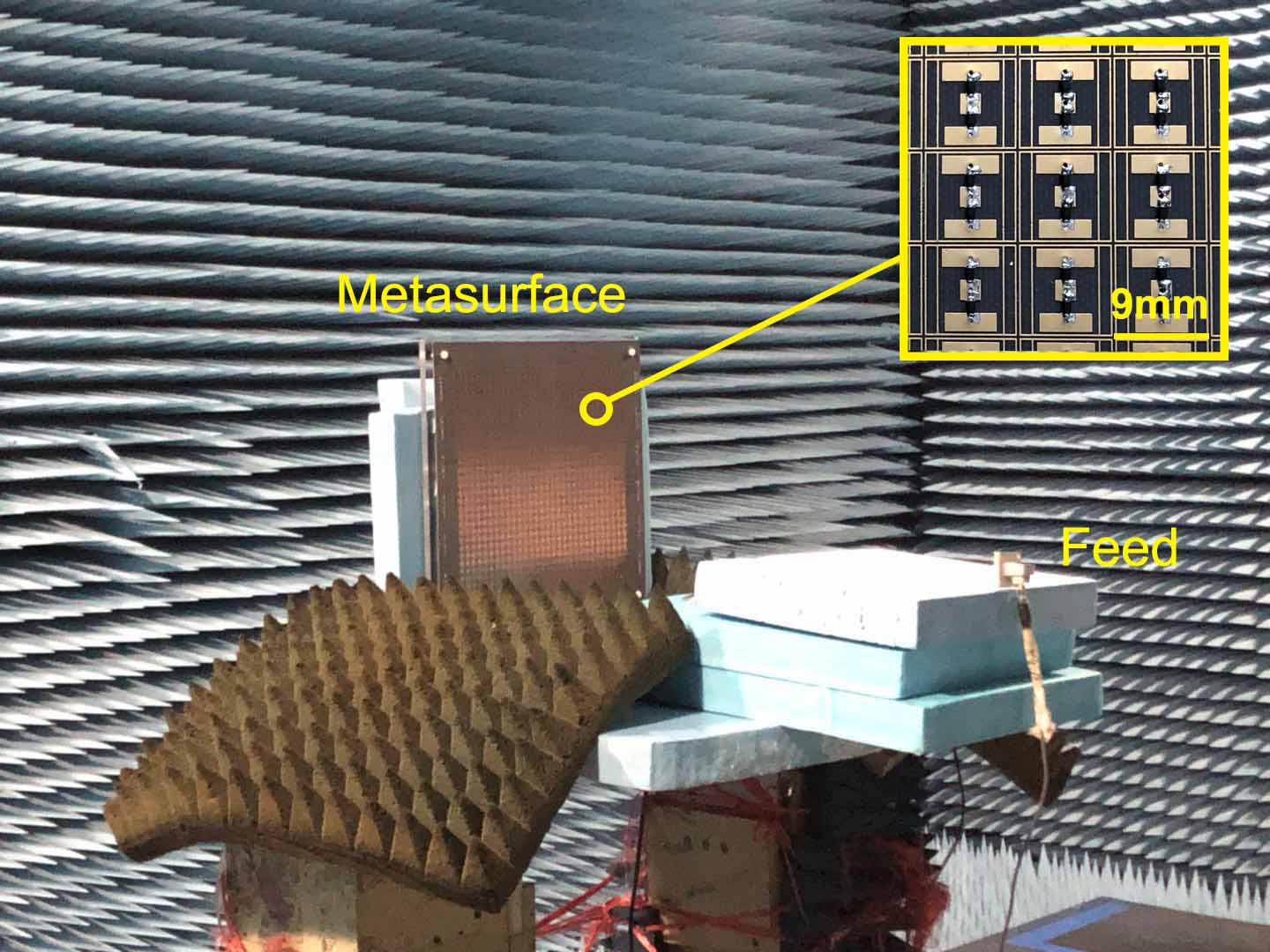


**Supplementary Figure S9.** Photograph of experimental setup and fabricated sample. The metasurface is fixed on the rotatable table to measure far-field power. The enlarged picture of the fabricated unit cells is provided.

**Note S9. The real-time display of sensor states**

In our smart metasurface platform, five kinds of sensors are integrated, including gyroscope, light sensor, height sensor, humidity sensor, and heat sensor. The sensing data collected by these sensors are all connected to MCU (Arduino UNO) for processing. The light sensor module is based on a photo-resistor (GL5516), which is sensitive to the visible light (380~780 nm). The humidity and heat sensor is DHT11 module. The height and air-pressure sensor is BOSCH BMP180. All sensors are arranged on the back of the metasurface, as shown in Fig. S10a. A display screen is applied here, to show the real-time states of all sensors. The detailed displayed data of each sensor are indicated in Fig. S10b, including the deflection angles in 3-axis, air pressure, light intensity, temperature, humidity, and height.


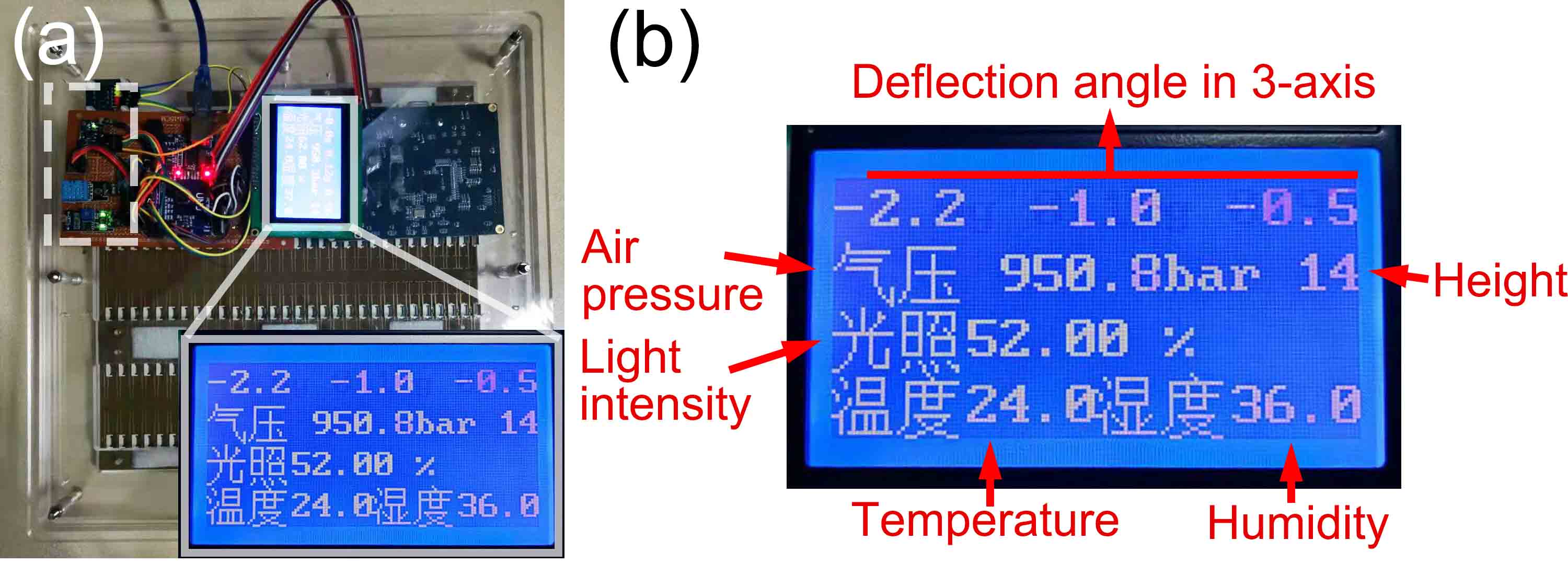


**Supplementary Figure S10.** (a) The assembled smart metasurface platform. (b) The real-time information on the screen of each sensor.

**Note S10. Coding patterns and far-field results for other functions**

Based on the smart metasurface, we can realize many other functions of beam controls when the metasurface rotates. As examples, Figures S11a-c show three representative functions. Since the rotation angle in the elevation direction (*phi*) is less than 45°, the metasurface will produce a single beam; when the rotation angle is between 45°~90°, the metasurface will generate OAM beam; and when the rotation angle is larger than 90°, the metasurface will produce diffuse scattering.

We mainly demonstrate Functions 2 and 3, since Function 1 is similar to the single-beam modualtion. The coding patterns for Functions 2 and 3 are shown in Figs. S11d and g. The simulated far-field results in the upper half space are provided in Figs. S11e and h for Functions 2 and 3, respectively. The related measurement results are presented in Figs. S11f and i, which successfully verified the performance on these functions. The measured far-field radiation pattern of the OAM beam generation is shown in Fig. S11f, in which the amplitude zero in the center is clearly observed. The near fields are also measured on a near-field plane that is 800mm in front of the metasurface at 9 GHz, and the measured phase pattern is presented in the inset of Fig. S11f, further showing the good performance. Fig. S11i gives the measured results of diffusion scattering, in which a RCS reduction better than -10.8dB is clearly observed. In both functions, the measured and simulated results have good agreements.


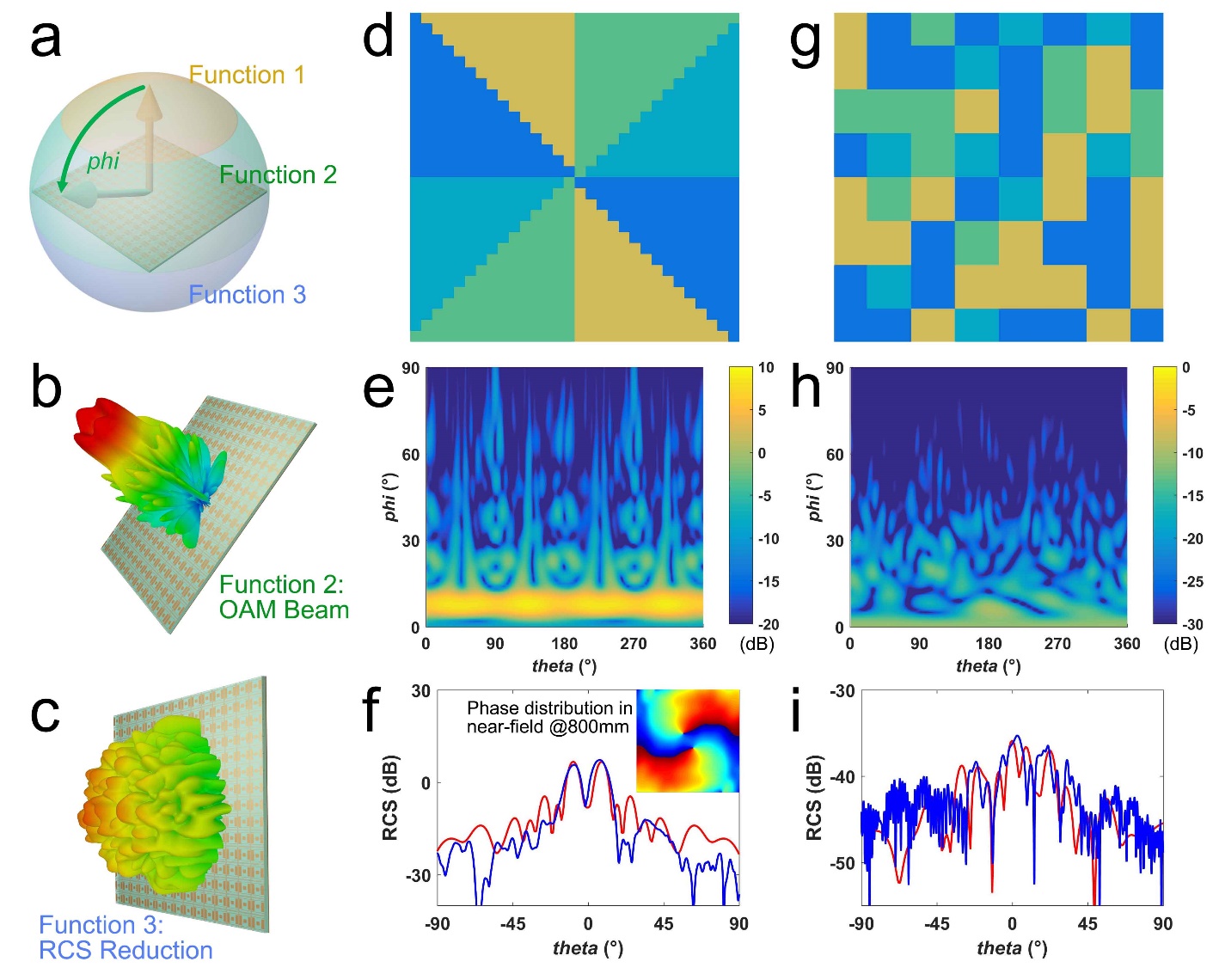


**Supplementary Figure S11.** The coding patterns and far-field results for Scheme B. (a) The illustration for Scheme B. (b) and (c) The scattering patterns of Functions 2 and 3: OAM beam generation and RCS reduction. (d) and (g) The coding patterns for the Function 2 and 3. (e) and (h) The simulated far-field radiation results in the upper half space. (f) and (i) The comparison between simulated and measured results in far-field, as well as the near-field phase distribution for Function 2. Here, the simulated and experimental results in far-field are marked with red and blue colors, respectively.
